# miR-1224 contributes to ischemic stroke-mediated natural killer cell dysfunction by targeting Sp1 signaling

**DOI:** 10.1186/s12974-021-02181-4

**Published:** 2021-06-12

**Authors:** Yan Feng, Yan Li, Ying Zhang, Bo-Hao Zhang, Hui Zhao, Xin Zhao, Fu-Dong Shi, Wei-Na Jin, Xiao-An Zhang

**Affiliations:** 1 Department of Neurology, Tianjin Medical University General Hospital, Tianjin, China; 2 China National Clinical Research Center for Neurological Diseases, Advanced Innovation Center for Human Brain Protection, Beijing Tiantan Hospital, Capital Medical University, Beijing, China; 3 The Third Affiliated Hospital of Zhengzhou University, No. 7 Kangfu front ST, Zhengzhou, Henan China; 4 School of Basic Medical Sciences, Henan University of Science and Technology, Luoyang, Henan China

**Keywords:** Ischemic stroke, Natural killer cells, microRNA, Immune suppression, Poststroke infection

## Abstract

**Background:**

Brain ischemia compromises natural killer (NK) cell-mediated immune defenses by acting on neurogenic and intracellular pathways. Less is known about the posttranscriptional mechanisms that regulate NK cell activation and cytotoxicity after ischemic stroke.

**Methods:**

Using a NanoString nCounter® miRNA array panel, we explored the microRNA (miRNA) profile of splenic NK cells in mice subjected to middle cerebral artery occlusion. Differential gene expression and function/pathway analysis were applied to investigate the main functions of predicted miRNA target genes. miR-1224 inhibitor/mimics transfection and passive transfer of NK cells were performed to confirm the impact of miR-1224 in NK cells after brain ischemia.

**Results:**

We observed striking dysregulation of several miRNAs in response to ischemia. Among those miRNAs, miR-1224 markedly increased 3 days after ischemic stroke. Transfection of miR-1224 mimics into NK cells resulted in suppression of NK cell activity, while an miR-1224 inhibitor enhanced NK cell activity and cytotoxicity, especially in the periphery. Passive transfer of NK cells treated with an miR-1224 inhibitor prevented the accumulation of a bacterial burden in the lungs after ischemic stroke, suggesting an enhanced immune defense of NK cells. The transcription factor Sp1, which controls cytokine/chemokine release by NK cells at the transcriptional level, is a predicted target of miR-1224. The inhibitory effect of miR-1224 on NK cell activity was blocked in Sp1 knockout mice.

**Conclusions:**

These findings indicate that miR-1224 may serve as a negative regulator of NK cell activation in an Sp1-dependent manner; this mechanism may be a novel target to prevent poststroke infection specifically in the periphery and preserve immune defense in the brain.

**Supplementary Information:**

The online version contains supplementary material available at 10.1186/s12974-021-02181-4.

## Introduction

Cerebral ischemia is a leading cause of mortality and severe neurological disability [1]. The impairment of systemic immune responses following brain ischemia is thought to protect the brain from further inflammatory insults, but it simultaneously increases susceptibility to infections, such as pneumonia and urinary tract infections [2]. Natural killer (NK) cells are innate lymphoid cells that are critical for host defense against infection. In a prior study, we demonstrated that neuroendocrine pathways inhibited NK cell responses in the central nervous system (CNS) and the periphery after ischemic stroke, and identified SOCS3- and RUNX3-mediated molecular pathways that were differentially modulated in NK cells [3]. Apart from transcriptional regulation, recent studies have suggested that posttranscriptional regulation, including the action of microRNAs (miRNAs), partially controls specialized NK cell effector functions, including activation as well as IFN-γ and granzyme production.

miRNAs are short (∼ 22 nt) endogenous noncoding RNAs that target protein-coding mRNAs for translational repression or degradation [4]. miRNAs are involved in a variety of physiological and pathological life processes [5] and play central roles closely associated with ischemic stroke, such as proliferation, hematopoiesis, metabolism, immune function, and immune depression after stroke [6]. In vivo mouse and human studies revealed temporal changes in miRNA expression during stroke progression, which may modulate several pathogenic processes that contribute to stroke etiology, including atherosclerosis (miR-21, miR-126), hyperlipidemia (miR-33, miR-125a-5p), hypertension (miR-155), and plaque rupture (miR-222, miR-210) [7]. Further studies reported that miRNAs can be used as prognostic, diagnostic, and therapeutic biomarkers of stroke [8, 9].

miRNAs have been demonstrated to play an indispensable role in the innate immune response by regulating leukocyte development [10, 11]. Previous studies using individual gain- and loss-of-function miRNAs in NK cells have demonstrated the roles of specific miRNAs in regulating NK cell development, maturation, and activation [12–17]. miRNAs also regulate fundamental NK cell processes such as cytokine production, cytotoxicity, and proliferation [18]. Huang et al. reported that overexpression of miR-30e inhibited cytotoxic activity in NK cells activated by IFN-α [19]. Xu et al. found that miR-146a exhibited a negative regulatory effect on NK cell functions by targeting STAT1 and that miR-146a could be induced by anti-inflammatory cytokines, such as IL-10 and TGF-β [20]. However, it is still unclear whether miRNAs are involved in NK cell phenotype variation after ischemic stroke and especially whether these molecules contribute to poststroke immunosuppression. Using RNA sequencing technology, we identified a variety of miRNAs that were upregulated in NK cells after ischemic stroke. The most promising candidate was miR-1224, which inhibited the activation and cytotoxicity of NK cells through the Sp1/IFN-γ signaling pathway in the periphery. Sp1 belongs to the specificity protein (Sp)/Kruppel-like transcription factor family, a group of proteins that recognize G-rich promoter elements (the GC box and the related GT box) and are expressed in most mammalian cell types. The Sp1 transcription factor is thought to regulate generic processes such as the cell cycle and growth control, metabolic pathways, and apoptosis [21]. Our data demonstrated that targeting miR-1224 in NK cells may be a novel strategy to potentiate immune defense in the periphery and prevent poststroke infection through a mechanism that depends on Sp1 signaling.

## Materials and methods

### Animals

Male C57BL/6 mice were purchased from Taconic (Oxnard, CA, USA). NOD-Prkdc^scid^ IL2rg^−/−^ mice (stock no. VS-AM-001) were provided by Vitalstar Biotechnology (Beijing, China) [22]. Heterozygous Sp1-knockout mice were purchased from GemPharmatech (Wilmington, DE, USA). Briefly, sgRNA was transcribed in vitro. Cas9 and sgRNA were microinjected into the fertilized eggs of C57BL/6 mice. Fertilized eggs were transplanted to obtain positive F0 mice, whose genotype was confirmed by PCR and sequencing. A stable F1 generation mouse model was obtained by mating positive F0 generation mice with C57BL/6 mice. Age-matched, 10- to 12-week-old male littermates weighing 20 to 25 g were used in each experimental group. All mice were housed in standard conditions at 22.2 °C with a 12/12-h light/dark cycle and had ad libitum access to food and water. All experiments were conducted in accordance with the Animal Research: Reporting of In Vivo Experiments (ARRIVE) guidelines (https://www.nc3rs.org.uk/arrive-guidelines) [23] and approved by the Committee on the Ethics of Animal Experiments of Tianjin Neurological Institute and Tianjin Medical University (Tianjin, China).

### Induction of middle cerebral artery occlusion mouse model

Middle cerebral artery occlusion (MCAO) was induced in 10- to 12-week-old mice as described previously [24, 25]. Animals were anesthetized by intraperitoneal injection of 5% chloral hydrate (30 mg/kg). A heating blanket was used throughout surgery to maintain the body temperature of the animals at 37.0 ± 0.5 °C until they awoke from anesthesia. The left common carotid artery, external carotid artery, and internal carotid artery were exposed through an incision and then isolated and ligated. A 5–0 nylon monofilament was inserted through the common carotid artery into the internal carotid artery and advanced to the beginning of the middle cerebral artery. The occlusion and reperfusion of the middle cerebral artery were monitored with a laser Doppler blood flowmeter (model P10, Moor Instruments, Wilmington, DE, USA) positioned 1 mm posterior and 3 mm to the left of bregma. After 60 min of ischemia, the nylon monofilament was removed to restore blood flow. We included only mice that had a residual cerebral blood flow (CBF) < 15% throughout the ischemic period and recovered > 80% of baseline CBF within 10 min of reperfusion. Sham control mice were subjected to the same surgical procedure, except that the nylon monofilament was not advanced far enough to occlude the middle cerebral artery.

### Neurological assessment

Behavior tests were performed on day 1 and day 3 post-MCAO by two investigators who were blinded to the experimental group assignment; the procedures were as previously described [26, 27]. The modified neurological severity score (mNSS) was used to evaluate sensory and motor function, reflexes, and balance. After recovering from MCAO surgery, each mouse was assessed on a scale from 0 to 18. The higher the score, the more severe the impairment was. The corner test was used to evaluate sensorimotor and postural asymmetries. All the mice were allowed to enter a 30° corner and then freely turn either left or right to exit the corner. The choice of direction was recorded in 10 trials, and the percentage of left turns was calculated. The rotarod test aims to assess systemic motor function. Every mouse was trained 3 days before MCAO surgery and tested 3 times daily after MCAO. The speed was increased from 4 to 40 rpm at an acceleration rate of 20 rpm/min and then continued at 40 rpm, for a total test duration of up to 10 min. The latency to fall off the rotating rod was recorded by an investigator who was blinded to the experimental treatments, and the mean of three trials was used for analysis.

### Neuroimaging

The infarct size in the MCAO model was evaluated with a 7-T small-animal MRI scanner as previously described (Bruker Daltonics Inc., Billerica, MA, USA) [3]. Anesthesia was induced with 5% isoflurane and maintained with 1.0–2.0% isoflurane in 70% N_2_O and 30% O_2_. Mice were placed on a heat-regulated blanket to maintain their body temperatures at 37.0 ± 0.5 °C during the scans. T2-weighted images of the brain were used to detect the infarct size in the MCAO mouse model; these images were obtained with a fat-suppressed rapid acquisition with relaxation enhancement sequence (repetition time = 4000 ms, echo time = 60 ms, field of view = 19.2 × 19.2 mm^2^, matrix size = 192 × 192, slice thickness = 0.5 mm). The MRI data were analyzed with ImageJ software (National Institutes of Health, Bethesda, MD, USA).

### Isolation of NK cells

NK cells were sorted from spleens on days 1, 3, and 7 after MCAO or the sham operation as previously described [3]. For RNA sequencing, NK cells were isolated via flow cytometry with high purity (≥ 99%) and viability. For adoptive transfer, magnetic-bead sorting system was used to enrich the NK cells in a cell suspension from the spleens of MCAO mice or sham-operated mice by applying anti-NK1.1 microbeads (Miltenyi Biotec, San Diego, CA, USA). Fluorescence-activated cell sorting (FACS) was used to analyze isolated cells and to determine the efficiency of NK cell sorting and the purity of the products.

### miRNA expression

NK cells were sorted from spleens on days 1, 3, and 7 after MCAO or the sham operation as previously described [3]. TRIzol Reagent (Thermo Fisher Scientific, Waltham, MA, USA) and a total RNA isolation kit (Qiagen, Germantown, MD, USA) were used according to the manufacturer’s protocol to isolate total RNA from NK cells pooled from 15 mice per data point. Then, the RNAs were analyzed with an nCounter miRNA Expression Assay (NanoString Technologies, Inc., Seattle, WA, USA), which can detect multiple miRNAs.

### Normalizing and analyzing NanoString data

Raw miRNA data were analyzed by nSolver Analysis Software v.3.0 (NanoString Technologies, Inc.). Data were normalized by using positive and negative controls and housekeeping gene probes. After all relevant correction and normalization steps were performed, statistical tests were applied to identify differentially expressed miRNAs. The resulting p values were adjusted by using the false discovery rate (FDR) procedure. miRNAs with an FDR value of < 0.05 and a fold change of > 2.0 were considered to be significantly differentially expressed.

### Prediction of miRNA target genes and construction of the miRNA-mRNA network

The miRNA target prediction tools TargetScan and miRanda were utilized to further explore the target mRNAs that were regulated by differentially expressed miRNAs. By combining the differentially expressed miRNAs and mRNAs as well as the predicted targets for these miRNAs, a core miRNA-mRNA regulatory network was constructed by using Cytoscape software.

### GO analysis and KEGG pathway analysis

Gene Ontology (GO) analysis [28] was applied to analyze the main functions of the specific genes with significant differences in the representative profiles of miRNA target genes. Fisher’s exact test was applied to determine the significant GO categories, and p values were corrected by FDR. Only GO terms that had p values of < 0.001 and FDR values of < 0.05 were chosen. Enrichment provides a measure of the specificity of a function: the greater the enrichment, the more specific the corresponding function is, which helped us identify the GO terms with the most concrete functional descriptions in the experiment.

Pathway analysis was used to identify the significant pathways of the differentially expressed genes [29]. Pathway annotations of microarray genes were downloaded from the Kyoto Encyclopedia of Genes and Genomes (KEGG, http://www.genome.jp/kegg/). Fisher’s exact test was also used to identify the significantly enriched pathways. The resulting p values were adjusted using the Benjamini–Hochberg FDR algorithm. Pathway categories with p values < 0.05 are reported.

### Real-time PCR

Quantitative real-time PCR was performed as previously described [3]. Total RNA was extracted from NK cells with TRIzol Reagent (Thermo Fisher Scientific, Waltham, MA, USA) and a total RNA isolation kit (Qiagen). Then, cDNA from sorted NK cells was synthesized with a SuperScript III First Strand cDNA Synthesis kit (Invitrogen, Carlsbad, CA, USA) and analyzed by normalizing the expression of the gene of interest to GAPDH. Quantitative real-time PCR was performed using SsoAdvanced™ SYBR® Green Supermix (Bio-Rad, Hercules, CA, USA) on the CFX96 Real-time PCR Detection System (Bio-Rad).

### Synthesis of miR-1224 mimics and inhibitors

The miR-1224 mimics and inhibitors, as well as a negative control of miRNA, were synthesized by GenePharma (Shanghai, China) with the following sequences: miR-1224 mimics (5′-GUGAGGACUGGGGAGGUGGAG-3′, R: 5′-CCACCUCCCCAGUCCUCACUU-3′), miR-1224 inhibitors (5′-CUCCACCUCCCCAGUCCUCAC-3′), and negative control (5′-UUCUCCGAACGUGUCACGUTT-3′).

### Transfection and passive transfer of NK cells

After being stained with anti-NK1.1 microbeads, NK cells were sorted by a direct magnetic cell labeling system (Miltenyi Biotec) from the pooled splenocytes of wild-type mice. Next, NK cells were cultured in RPMI medium (Gibco, Grand Island, NY, USA) with 10% FBS (Gibco) and 1% penicillin/streptomycin (Solarbio, Beijing, China) and then transfected with 100 pmol miR-1224 mimics, inhibitor, or negative control using Lipofectamine 2000 (Invitrogen) according to the manufacturer’s instructions. After transfection, NK cells were cultured in vitro at 37 °C for 24 h, and the transfected NK cells were then injected into NOD-Prkdcscid IL2rg^−/−^ mice via the tail vein before MCAO model induction.

### Flow cytometry

Single-cell suspensions were prepared from the spleen or digested from the brain and stained with fluorochrome-conjugated antibodies. Antibodies against the following antigens were used: CD45 (30-F11), CD3 (145- 2C11), NK1.1 (PK136), NKG2A (18d3), KLRG1 (2F1), NKG2D (CX5), CD25 (PC61), CD69 (H1.2F3), IFN-γ (XMG1.2), and perforin (S16009A). Flow cytometry data were collected with a FACSAria III (BD Biosciences, San Jose, CA, USA) and analyzed by FlowJo 7.6 software (Informer Technologies, Walnut, CA, USA).

### Microbiologic analyses (CFU assay)

In order to observe lung infection after stroke, lung tissue was collected using aseptic techniques under sterile conditions on day 3 after MCAO. Lungs were ground with a pestle at a laminar flow bench to form a homogenate and serially diluted with PBS. The dilutions ranged from 1:5 to 1:1000. All procedures were performed in a sterile environment to avoid bacterial contamination. Agar plates (Solarbio) were inoculated with gradient concentrations of lung tissue homogenate and incubated for 24 h in a 37 °C incubator. Then, the growth colonies were counted by the following method: CFU/mL = (no. of colonies × dilution factor)/volume of culture plate.

### Statistical analysis

All data were analyzed by investigators who were blinded to all groups. No statistical methods were used to predetermine sample sizes, but our sample sizes were similar to those reported in previous publications. Animals were randomly assigned to experimental groups. Statistical analysis was conducted on data from three or more biologically independent experimental replicates. Data are shown as the mean ± SEM. Mean values were compared using Student’s t-test for comparisons between 2 groups and 1-way or 2-way repeated-measures ANOVA with a post hoc Bonferroni test for comparisons among more than 2 groups. p values < 0.05 were taken to indicate statistically significant differences. All statistical analyses were performed using GraphPad Prism 8.0 software (GraphPad, San Diego, CA, USA).

## Results

### miRNA profiling in peripheral NK cells after ischemic stroke

**Fig. 1:**
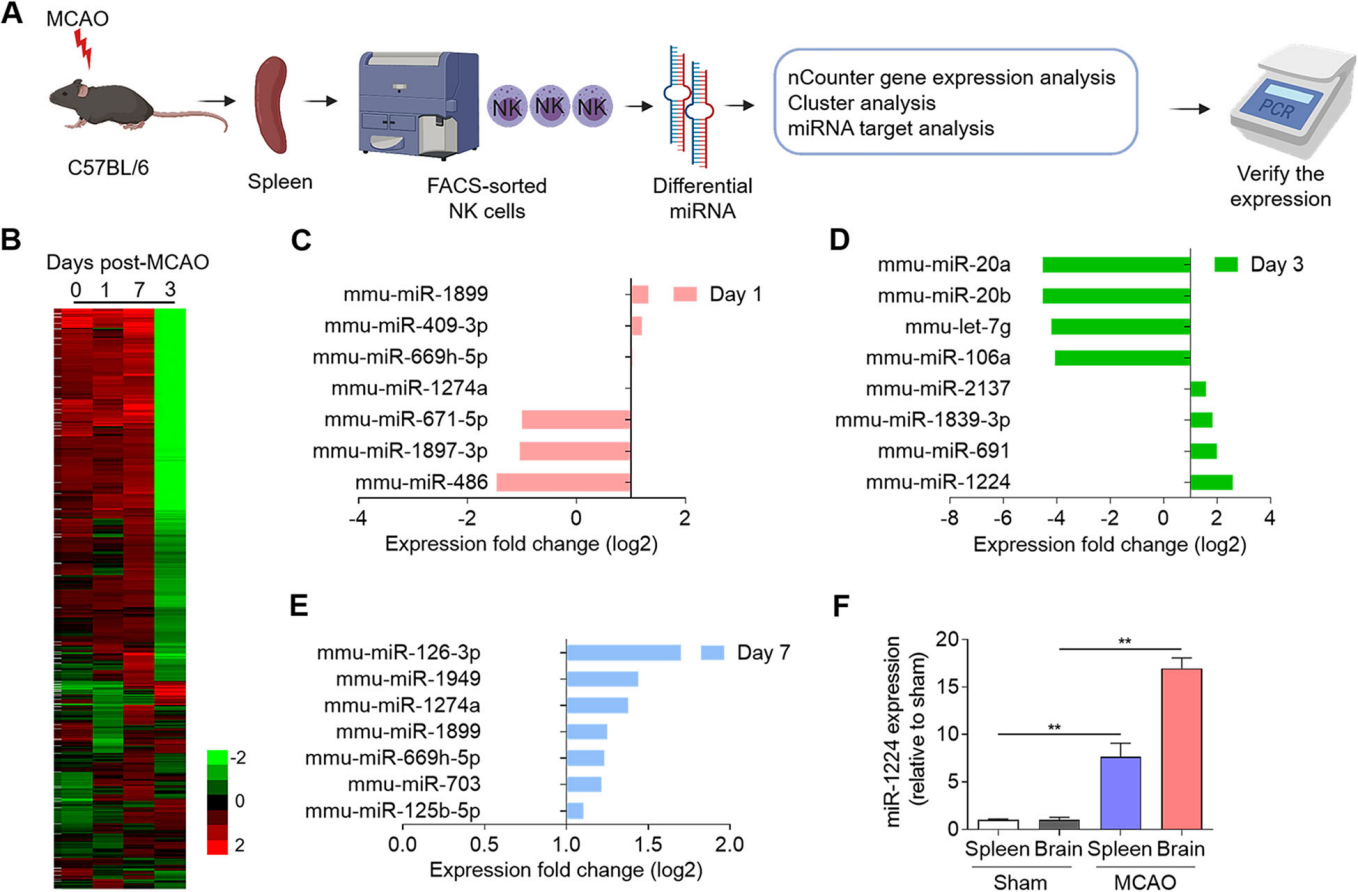
miRNA profile in splenic NK cells of C57BL/6 mice subjected to MCAO. **a** Technology roadmap of NK cell isolation and profiling. Splenic NK cells isolated from C57BL/6 mice after MCAO. RNA sequencing to identify miRNA signatures and reverse validation by RT-PCR. **b** The heatmap shows the quantitative nCounter expression profiling of 270 miRNAs in NK cells from the spleens of sham-operated or MCAO mice on days 1, 3, and 7 after surgery. The relative abundance of transcripts is indicated by the color (red, high; green, low). Data were pooled from 15 mice in each group. **c**–**e** The bar graph shows selected miRNAs that were upregulated or downregulated in NK cells from the spleen on postoperative days 1, 3, and 7 in MCAO mice compared to control (sham-operated) mice. **f** Verification of miR-1224 expression in NK cells from the spleens and brains of sham-operated or MCAO mice on postoperative day 3. Data are representative of three independent experiments. n = 8 mice per group. **p < 0.01 by one-way ANOVA. Error bars represent the mean ± SEM

The NanoString nCounter platform was used to identify miRNA signatures in MCAO mice. Spleens from 15 mice per group were pooled, and NK cells were sorted by flow cytometry. The small RNAs were extracted and analyzed by RNA sequencing (NanoString nCounter) (Fig. 1a). Global miRNA profiling revealed upregulation of 34 and downregulation of 236 miRNAs in peripheral NK cells of MCAO mice compared to the sham group on poststroke days 1 to 7 (Fig. 1b, unpaired t-test, Benjamini–Hochberg FDR < 0.05, fold change > 2, p < 0.05). When comparing the miRNA levels between different time points, we observed upregulation of miR-409-3p, which participated in inflammatory cytokine production, on the first day after reperfusion [30] (Fig. 1c, p < 0.05). Many miRNAs were downregulated on day 3, suggesting that suppressive posttranscriptional regulation by miRNAs occurred mainly in the subacute stage after brain ischemia. miR-1224 and miR-691, which have been reported to inhibit cell proliferation [31], were significantly increased on day 3 (Fig. 1d, p < 0.05). Most miRNAs were upregulated on day 7 after MCAO, including miR-126-3p, miR-1949, miR-1274a, miR-1899, miR-669h-5p, miR-703, and miR-129-5p (Fig. 1e; p < 0.05). Real-time PCR was then performed to verify miRNA expression in NK cells, revealing that miR-1224 was significantly increased in both the spleen and the brain on day 3 after MCAO (Fig. 1f; p < 0.05). These results displayed miRNA signatures in peripheral NK cells after brain ischemia.

### GO and KEGG pathway analysis of putative miRNA target genes

**Fig. 2:**
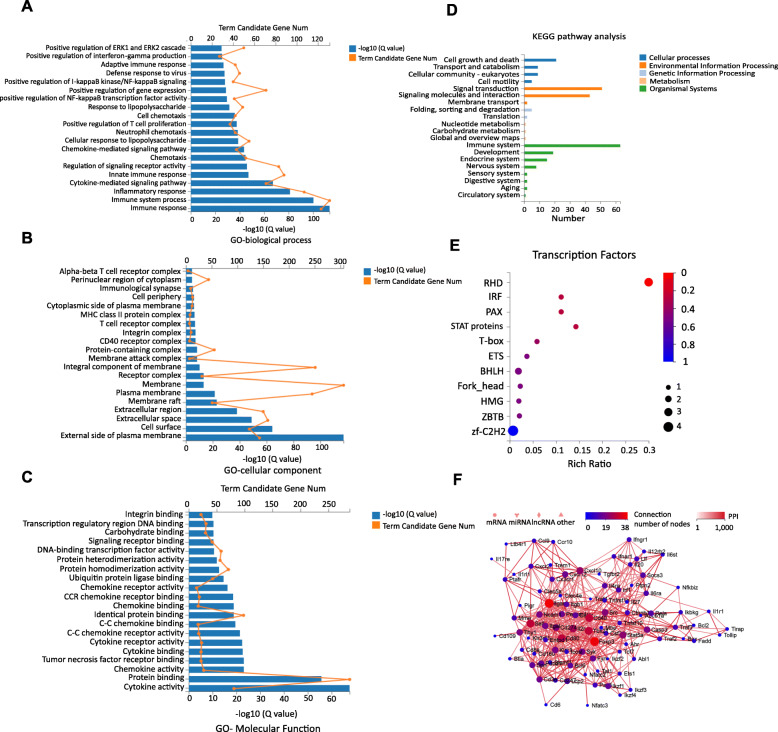
Enriched GO and KEGG of miRNAs and miRNA-mRNA interaction networks in NK cells after MCAO. Top 20 processes revealed by GO enrichment analysis to influence biological processes (**a**), cellular components (**b**), and molecular functions (**c**). **d** KEGG enrichment analysis results of all miRNA target genes. **e** Analysis of the regulatory networks of transcription factors for miRNA target genes. **f** Core miRNA-mRNA regulatory network. RHD, proteins containing the Rel homology domain (RHD) are eukaryotic transcription factors; IRF, interferon regulatory factors are proteins that regulate the transcription of interferons; PAX, paired box domain; STAT proteins, signal transducer and activator of transcription (STAT) proteins are a family of transcription factors that are specifically activated to regulate gene transcription when cells encounter cytokines and growth factors; T-box, a group of transcription factors involved in limb and heart development; ETS, in the field of molecular biology, the E26 transformation-specific (ETS) family is one of the largest families of transcription factors and is unique to animals; bHLH, a basic helix-loop-helix (bHLH) is a protein structural motif that characterizes one of the largest families of dimerizing transcription factors; Fork_head, the fork head domain is a type of protein domain that is often found in transcription factors; its purpose is to bind DNA; HMG, high-mobility group (HMG) box, HMG box domains are involved in binding DNA and may also be involved in protein-protein interactions; ZBTB, the BTB (for BR-C, ttk and bab) or POZ (for pox virus and zinc finger) domain is present near the N-terminus of a fraction of zinc finger proteins and in proteins that contain the Kelch motif, as well as a family of pox virus proteins; zf-C2H2, zinc finger, C2H2 type

In order to explore the relationship between differentially expressed miRNAs and poststroke NK cell functions, Gene Ontology (GO) and KEGG pathway analysis were applied to the respective target genes of the miRNAs. GO analyses of representative profiles of miRNA target genes are shown in Fig. 2a–c. At the cellular component level, GO terms were mainly involved in the plasma membrane and cell surface. At the molecular function level, proteins participating in cytokine activity and protein binding functions were enriched. At the biological process level, the immune response was significantly enriched. According to the KEGG pathway database, we identified several signaling pathways related to signal transduction pathways and the TF regulatory network in post-MCAO NK cells, including cytokine-receptor interaction, the Th17 and Jak-Stat signaling pathways, and the Rel/NF-κB and NFAT families of transcription factors (Fig. 2d, e). Next, we built a network of genes according to the relationship among miRNA target genes and putative upstream regulatory molecules (Fig. 2f). We found that miR-1224 was one of the miRNAs regulating the most target genes; its targets included Casp3, Cd40, Foxp3, Il2ra, Itgam, Itgb1, Sell, Sp1, Src, and Stat5a, which are also hub genes in miRNA-mRNA regulatory networks. This predicted miRNA target gene network was in agreement with the data from our gene difference analysis, suggesting that miR-1224 may play a key role in NK cell properties after brain ischemia.

### miR-1224 negatively regulates NK cell activation and cytotoxicity after ischemic stroke

**Fig. 3:**
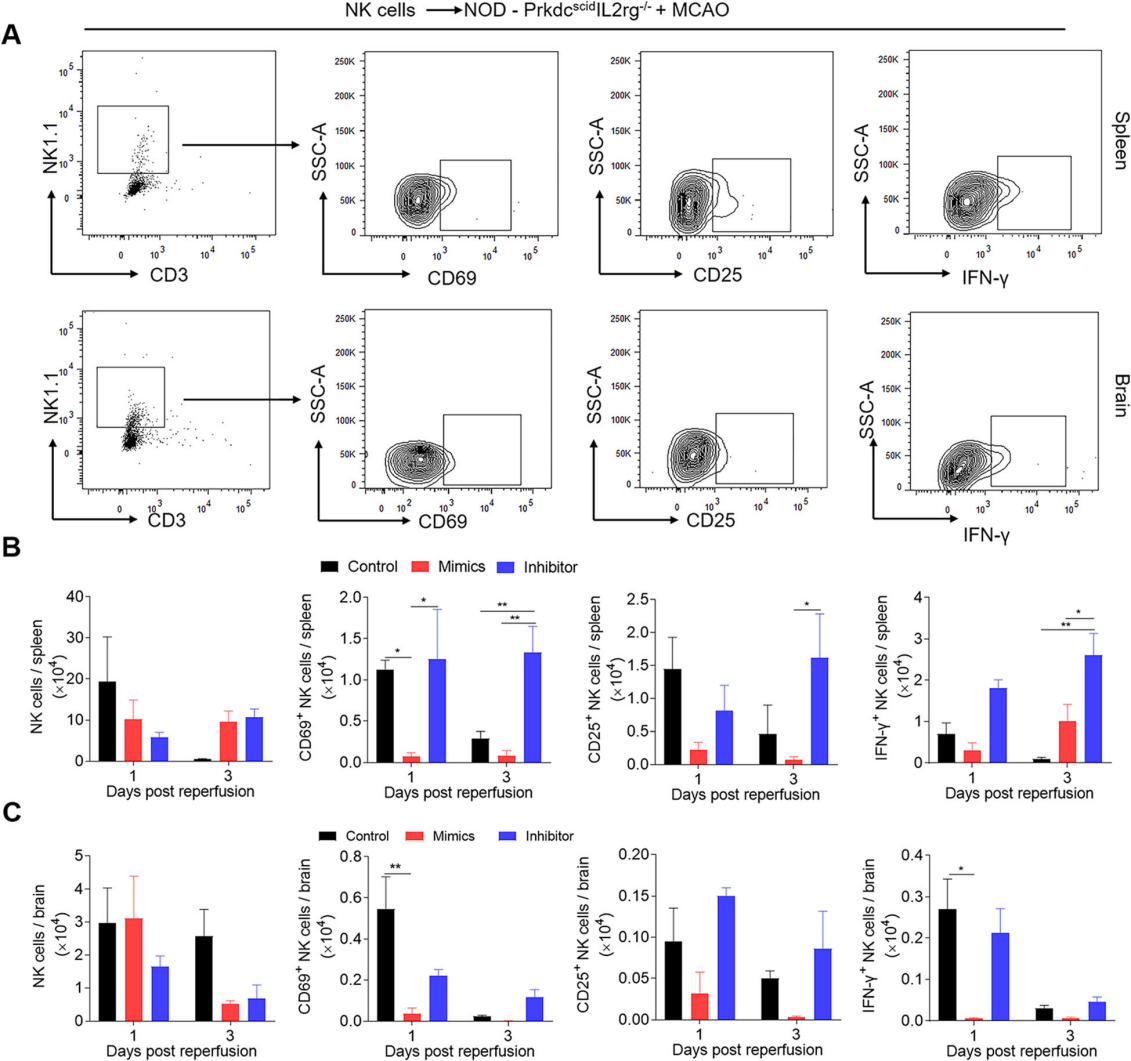
miR-1224 inhibited the activation and cytotoxicity of NK cells from spleens after ischemic stroke. **a** Contour plots of flow cytometry show gating strategies and the expression of NK1.1, CD69, CD25, and IFN-γ from the spleens and brains of NOD-Prkdcscid IL2rg^−/−^ transgenic mice (lack of T, B, and NK cells) subjected to MCAO. **b**, **c** Bar graphs summarizing the effect of miR-1224 modulation on NK cell counts and the expression of CD69, CD25, and IFN-γ in splenic (**b**) and brain-infiltrating NK cells (**c**). Data are representative of three independent experiments. n = 11 mice per group. *p < 0.05, **p < 0.01 by two-way ANOVA. Error bars represent the mean ± SEM

The above data suggested that miR-1224 was the most significantly increased miRNA 3 days after ischemic stroke. We hypothesized that miR-1224 might contribute to NK cell suppression during the subacute stage in an MCAO model. To verify the impact of miR-1224 in NK cells after brain ischemia, we modulated miR-1224 via an inhibitor or mimics followed by a passive transfer assay, which enabled us to focus specifically on the role of miR-1224 in NK cell activation and cytotoxicity without affecting other cell types. Splenic NK cells were isolated from wild-type C57BL/6 mice and transfected with miR-1224 mimics or inhibitor or a negative control in vitro. The miR-1224-modified NK cells were then transferred intravenously to NOD-Prkdc^scid^ IL2rg^−/−^ transgenic mice (lack of T, B, and NK cells) prior to MCAO surgery. NK cell counts, activation (CD69, CD25, NKG2A, NKG2D, KLRG1), and cytotoxicity (NK-derived IFN-γ and perforin) features were analyzed on post-MCAO days 1 and 3 by flow cytometry (Fig. 3, Supplementary Figure 1 and Supplementary Tables 1 and 2).

In the spleen, adoptive transfer of miR-1224-mimic NK cells produced a suppressed phenotype from day 1 to 3 after ischemia, including decreased cell counts, downregulation of the activation marker CD69, and upregulation of the inhibitory receptor KLGR1. However, NK cells treated with an miR-1224 inhibitor displayed an activated phenotype, as shown by increased CD69 and CD25 levels and enhanced cytotoxicity (IFN-γ and perforin), especially on post-MCAO day 3 (Fig. 3b, Supplementary Figure 1B and Supplementary Table 1). In the brain, miR-1224 transiently suppressed CD69 expression and IFN-γ release at day 1 after MCAO but did not significantly impact NK cell counts or NK cell activation after day 1 of brain ischemia (Fig. 3c, Supplementary Figure 1C and Supplementary Table 2). These results suggested that miR-1224 suppressed NK cell proliferation and activation, specifically in the periphery.

### miR-1224 negatively regulates NK cell function by modulating Sp1

**Fig. 4:**
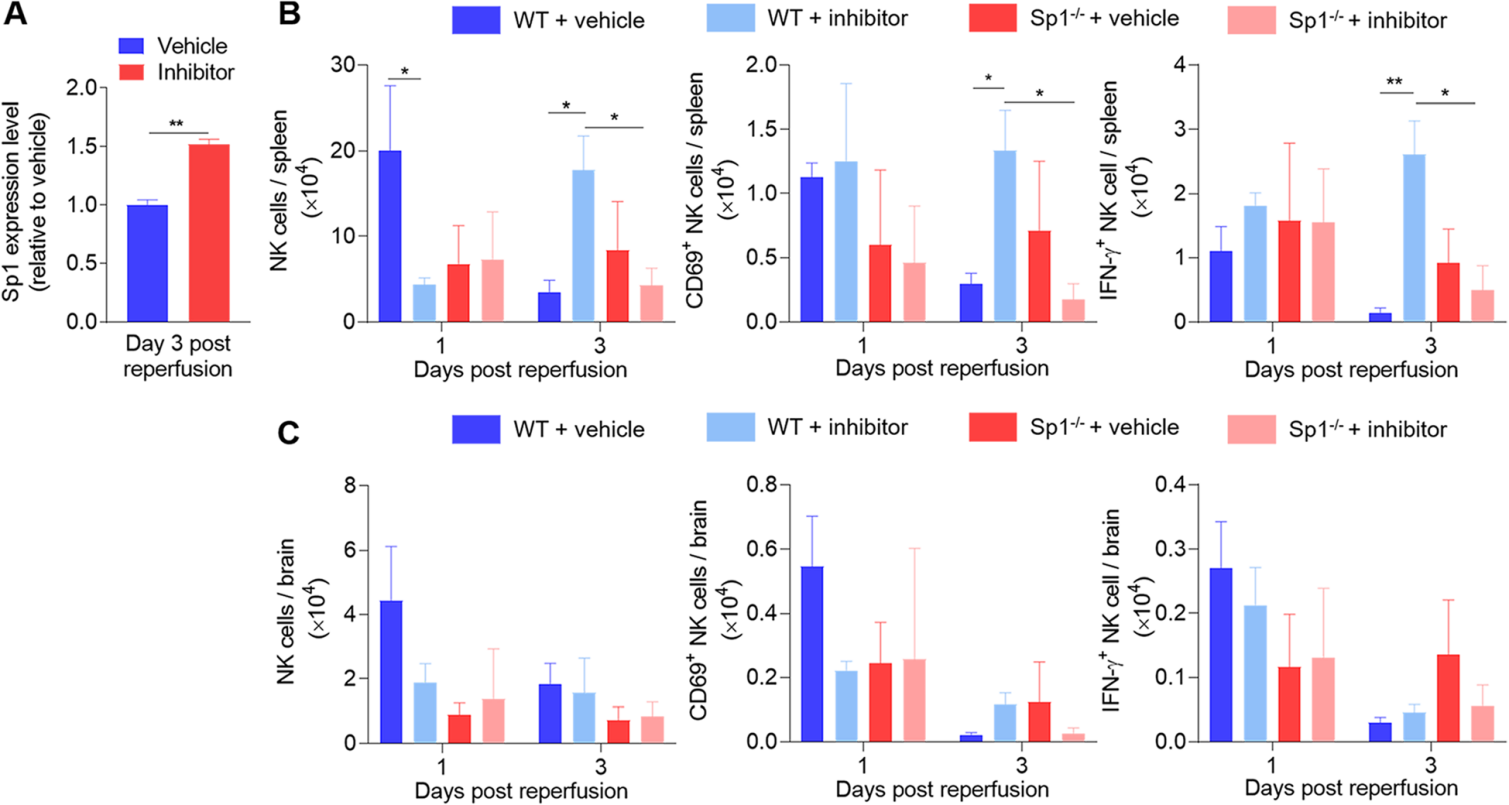
miR-1224 negatively regulates NK cell function in the spleen by modulating Sp1. **a** Sp1 gene expression was analyzed by real-time PCR after miR-1224 inhibitor treatment in MCAO mice. **b**, **c** Heterozygous Sp1-knockout mice were used to explore the role of the Sp1 transcription factor in NK cell regulation by miR-1224. Flow cytometry shows the numbers and functional markers (CD69, IFN-γ) of NK (NK1.1^+^CD3^−^) cells from the spleens and brains of wild-type and Sp1^−/−^ mice subjected to MCAO. Data are representative of three independent experiments. n = 3~11 mice per group. *p < 0.05, **p < 0.01 by two-way ANOVA. Error bars represent the mean ± SEM

It has been reported that miR-1224 negatively modulates specificity protein 1 (Sp1) by binding to the 3′ untranslated region (UTR); Sp1 protein, in turn, controls IFN-γ and TNF-α expression at the transcriptional level [32]. NK cells transfected with an miR-1224 inhibitor were transferred to NOD-Prkdcscid IL2rg^−/−^ mice via tail vein injection. Subsequently, the mice were subjected to cerebral ischemia and reperfusion, and Sp1 gene expression was quantified on day 3 after MCAO induction. Three days after MCAO, the expression of the Sp1 gene was significantly higher in mice transfected with the miR-1224 inhibitor than in mice transfected with the control miRNA (Fig. 4a). We wished to further explore whether miR-1224 suppresses NK cell function through the Sp1 pathway after ischemic stroke. Starting from heterozygous Sp1-knockout mice, we found that the miR-1224 inhibitor enhanced splenic NK cell activation and IFN-γ release in wild-type mice but failed to control the NK cell response without Sp1 signaling after MCAO (Fig. 4b, Supplementary Table 3). However, we did not observe significant intergroup differences in the brain (Fig. 4c, Supplementary Table 4). These data suggest that Sp1 might be involved in regulating NK cell depression mediated by miR-1224.

### Targeting miR-1224 with an inhibitor in NK cells reduces the bacterial burden in the lungs after MCAO

**Fig. 5:**
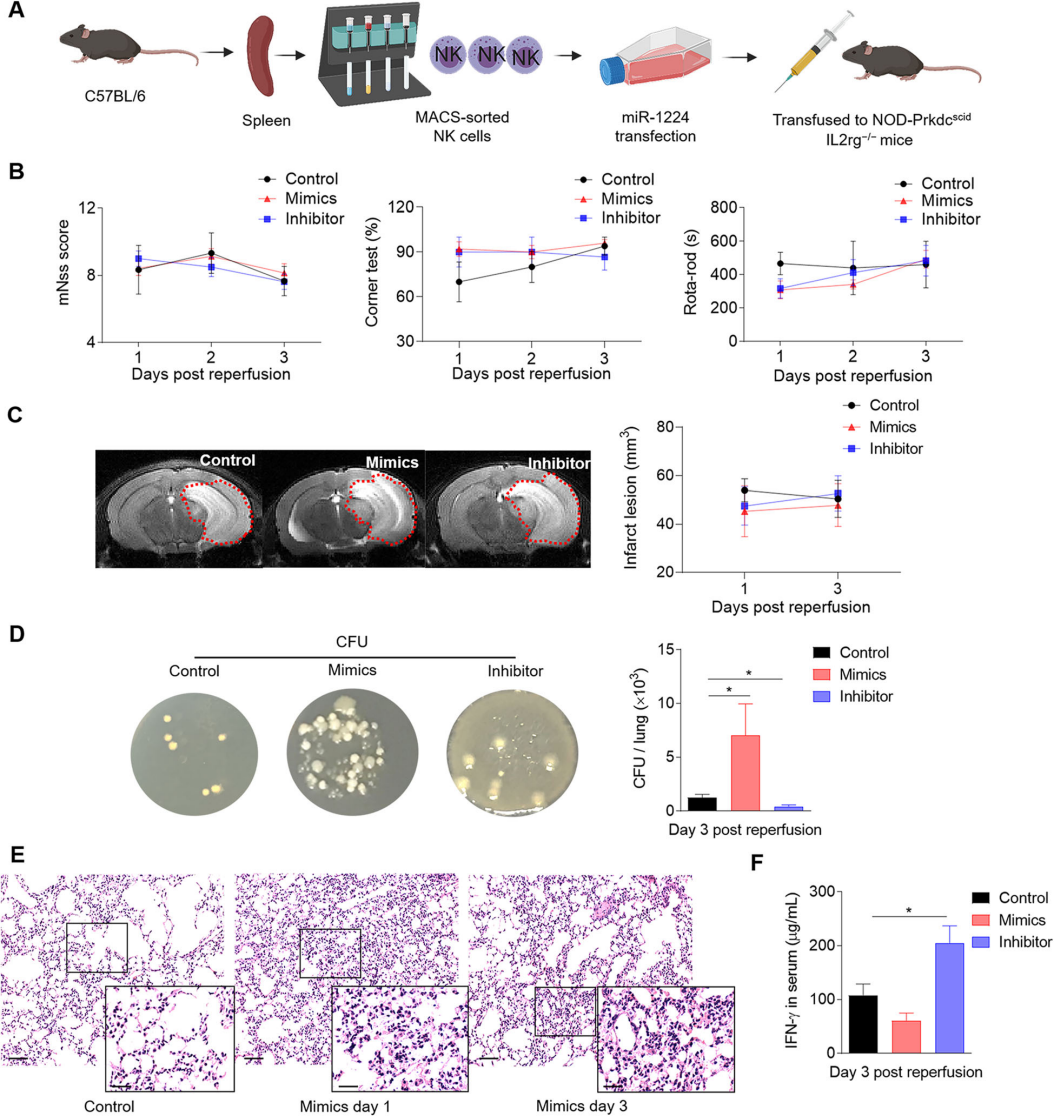
Downregulation of miR-1224 in NK cells by an inhibitor reduces the bacterial burden in the lungs of MCAO mice. **a** Schematic graph of the experimental design. Splenic NK cells isolated from C57BL/6 mice were transfected with miR-1224-mimics/inhibitor/negative control. miR-1224-regulated NK cells were then passively transferred via intravenous injection to NOD-Prkdc^scid^ IL2rg^−/−^ mice, followed by MCAO surgery. **b** Neurological deficits were assessed by the modified neurological severity score (mNSS), the corner test, and the rotarod test after NK cell transfer and MCAO surgery. n = 3~8 mice per group. p > 0.05 by two-way ANOVA. **c** Representative MR images and summarized data show stroke lesion volume after adoptive transfer of miR-1224-regulated NK cells; n = 3 mice per group. p > 0.05 by two-way ANOVA. **d** Lung tissues from MCAO mice treated with miR-1224-modulated NK cells were collected for bacteriological analysis 3 days after MCAO surgery. The bar graph illustrates the quantification of bacterial burden in the lungs of the indicated groups. Data are presented in colony-forming units (CFU) per lung tissue. n = 8 mice per group. *p < 0.05 by a two-tailed unpaired Student’s t-test. **e** NOD-Prkdc^scid^ IL2rg^−/−^ mice underwent adoptive transfer of NK cells transfected with an miR-1224 mimic plasmid or a control plasmid. Lung tissues were collected on post-MCAO day 3 for histological examination. The typical signs of bacterial burden (thickening of alveolar walls and neutrophilic infiltration) in MCAO mice were visible in lung sections stained with hematoxylin and eosin. Scale bars, 50 μm; insert, 25 μm. **f** The protein levels of IFN-γ in the serum of the control and mimic groups were measured by ELISA on post-MCAO day 3. n = 3~8 mice per group. *p < 0.05 by a two-tailed unpaired Student’s t-test. Data are representative of three independent experiments. Error bars represent the mean ± SEM

We next explored whether regulating miR-1224 in NK cells would impact the ischemic lesions and clinical outcomes after MCAO. miR-1224-modified (mimic-, inhibitor-, or control-transfected) NK cells were transferred intravenously to NOD-Prkdc^scid^ IL2rg^−/−^ mice, which were then subjected to MCAO surgery (Fig. 5a). A battery of sensorimotor behavioral tests were conducted, including mNSS scoring, the corner test, and the rotarod test. T2-weighted imaging was used to evaluate the infarct lesions on day 1 and day 3 after ischemic stroke. As shown in Fig. 5, targeting miR-1224 in NK cells with mimics or an inhibitor had no obvious effect on neurological function (Fig. 5b) or lesion size (Fig. 5c) from day 1 to 3 after ischemia.

The impairment of immune responses after brain ischemia increases susceptibility to lung infections [33, 34]. In this study, we aimed to determine whether miR-1224-mediated NK cell suppression contributes to poststroke infection. The colony-forming unit (CFU) assay was used to quantify bacterial load in animal models [35]. We collected lung tissue under sterile conditions, and any bacterial growth from this tissue was defined as spontaneous pulmonary infection. The CFU assay and histological staining of lung tissue revealed bacterial pathological changes on post-MCAO day 3, which were exacerbated compared to the control by the transfer of NK cells expressing miR-1224 mimics (Fig. 5d, e). With the transfer of NK cells expressing an miR-1224 inhibitor, we observed a reduction in bacterial colony counts and an increase in peripheral IFN-γ release on post-MCAO day 3 (Fig. 5f). This result indicates that the miR-1224 inhibitor may enhance the ability of NK cells to release IFN-γ and clear out bacteria in the periphery.

## Discussion

Several studies in a variety of diseases have helped to clarify the contribution of miRNAs to NK cell developmental intermediates and subsets, their role in tissues, and their significance in the context of disease. In the present study, we performed miRNA sequencing analysis on circulating NK cell populations isolated from an MCAO mouse model. Our data suggest that miR-1224 negatively regulates NK cell activation and IFN-γ release in an Sp1-dependent manner.

Pulmonary infection is a common complication of acute ischemic stroke. Two large international clinical trials revealed that prophylactic antibiotics did not prevent lung infection or improve neurological outcomes in patients with acute ischemic stroke [36, 37]. NK cells have been demonstrated to exacerbate brain infarction after ischemic stroke by promoting local inflammation and neuronal hyperactivity [38]. A previous study showed that patients in the acute phase of ischemic stroke (< 24 h) displayed significant atrophy of the spleen, as measured by magnetic resonance imaging (MRI). In addition to spleen size, the number of circulating NK cells was also significantly reduced. We have previously investigated the interaction between the nervous and immune systems in the context of brain ischemia, focusing on NK cells. We demonstrated that distinct neuroendocrine pathways inhibited NK cell responses in the CNS and the periphery and identified intracellular pathways that were differentially modulated in NK cells in the brain and spleen [3]. However, the current understanding of the basic molecular mechanisms regulating NK cell activation and function after ischemic stroke is still incomplete, especially at the posttranscriptional level. A number of studies have reported the expression of miRNAs by NK cells, their contribution to cell-intrinsic and cell-extrinsic control of NK cell development and effector response, and their dysregulation in NK cells during pathogenesis. For example, miR-27a-5p [39], miR-30e [40], and miR-150 [41] negatively regulate NK cell cytotoxicity by targeting perforin [40, 41], while miR-27a-5p targets both granzyme B and perforin [39]. In contrast, Ni and colleagues found that perforin, granzyme, IFN-γ, and CD107a in human NK cells were all upregulated after miR-362-5p overexpression, which means that miR-362-5p promotes NK cell effector functions [42]. Our present study identified a new target, miR-1224, which is one of the most important molecules mediating NK cell suppression after brain ischemia. We found that miR-1224 may suppress NK cell function through the Sp1 pathway after ischemic stroke. A related study demonstrated that miR-1224 decreased TNF-α by targeting the Sp1 protein in LPS-treated WT mice, which is in agreement with our finding [32]. Taken together, our results confirmed the posttranscriptional regulatory action of miR-1224 on NK cells after brain ischemia and suggested miR-1224–Sp1–IFN-γ signaling as its potential pathway.

In addition to the local inflammatory immune response in the brain, ischemic stroke strikingly alters systemic inflammation, leaving patients susceptible to immunosuppression and infections, which are related to poor functional outcomes and increased morbidity [43]. The impact of brain ischemia on the immune system has been documented mostly in peripheral lymphocytes [34, 44, 45], particularly in the spleen, which shrinks because of the apoptotic death of splenocytes and the migration of cells into the brain parenchyma [46]. In our study, we confirmed that miR-1224 suppressed NK cell activation and cytotoxicity specifically in the periphery rather than in the brain. Our data established that it is possible to enhance the cytotoxicity of peripheral NK cells by targeting miR-1224 while preserving the immunosuppression of brain-infiltrating NK cells to avoid aggravated intracerebral inflammation. This result is interesting and seemingly paradoxical because studies by our team and others have all suggested that NK cells can migrate into the brain parenchyma after brain ischemia. In our previous research, we found that distinct neuroendocrine pathways differentially inhibit NK cell responses in the central nervous system and the periphery after cerebral infarction [2, 3]. As the switching of neuroendocrine status is accompanied by key miRNA alterations [47], we speculate that crosstalk among neurogenic pathways, transcription, and posttranscriptional signaling may contribute to immune cell functions after ischemic stroke; this topic needs further investigation.

Our study has limitations that remain to be resolved in the future. First, although we found that miR-1224 may serve as a negative regulator of NK cell function after ischemic stroke, we cannot ignore the potential involvement of other miRNAs in the pathology of ischemic stroke. For example, although miR-1224 was the most promising candidate from our screening, miRNA-451a and miRNA-122-5p were also found to be upregulated in the miRNA array and have also been documented to have inhibitory effects on the activation of NK cells [48]. Another limitation of this study lies in the short lifetime of transfected cells: genetic response from NK cells can be detected only within a limited period after transfection. Such a transient response cannot faithfully represent the long-term change in NK cells after stroke. Therefore, future research will investigate more efficient modulation methods for long-term studies.

In summary, our study indicated that miR-1224 suppresses NK cell function through Sp1 after ischemic stroke, especially in the periphery. These results suggest that blocking miR-1224 biogenesis or administering a miR-1224 antagonist might be a viable therapeutic approach for poststroke immunosuppression and infection.

## Supplementary Information

**Additional file 1: Supplementary Figure 1.** The effect of miR-1224 on the phenotype and function of NK cells in the brain and periphery after ischemia and reperfusion. After treatment with miR-1224 mimics or inhibitor or a negative control, NK cells were transferred intravenously into NPG mice before MCAO. Cells were isolated from the spleen and brain at the indicated time points after surgery. A-C. Flow cytometry plots and summarized results show the influence of miR-1224 on NK cells in the spleen (B) and brain (C) in the acute stage of ischemic stroke, including inhibitory receptors on NK cells (NKG2A and KLRG1), activating receptors on NK cells (NKG2D), and the cytotoxic function of NK cells (perforin).

**Additional file 2: Supplementary Table 1.** miR-1224 inhibits the activation and cytotoxicity of splenic NK cells after ischemic stroke. Flow cytometry was used to analyze the number of functional markers in NK cells. Data are presented as the mean ± SEM. *p < 0.05, **p < 0.01 in mimics versus control; #*p* < 0.05, ##*p* < 0.01 in inhibitor versus control; &*p* < 0.05, &&*p* < 0.01 in inhibitor versus mimics. **Supplementary Table 2.** miR-1224 is involved in the alteration of brain-infiltrating NK cells after MCAO. Splenic NK cells isolated from wild-type mice were treated with an miR-1224 control, mimic or inhibitor plasmid. After transfection, NK cells were transferred intravenously into NPG mice before MCAO. The table shows the NK cell counts and the expression of functional markers of NK cells in the ischemic brain. Data are expressed as the mean ± SEM. *p < 0.05, **p < 0.01 in mimics versus control. **Supplementary Table 3.** miR-1224 negatively regulates NK cell function by modulating Sp1. Flow cytometry shows splenic NK cell counts and functional markers (CD69 and IFN-γ) of wild-type and Sp1-/- mice subjected to MCAO. Data were presented as the mean ± SEM. *p < 0.05 in WT/inhibitor versus WT/vehicle; #*p* < 0.05 in WT/inhibitor versus Sp1-/-/inhibitor. **Supplementary Table 4.** miR-1224 negatively regulates NK cell function by modulating Sp1. Flow cytometry shows brain-infiltrating NK cell counts and functional markers (CD69 and IFN-γ) in wild-type and Sp1-/- mice subjected to MCAO. Data and presented as the mean ± SEM.

## Data Availability

All data generated or analyzed during this study are included in this published article, its supplementary information files, and are available from the corresponding author on reasonable request.

## Abbreviations

CNS: Central nervous system
CFU: Colony-forming unit
CBF: Cerebral blood flow
C57BL/6: C57 black 6, a wild-type mouse strain used to generate mouse models
FDR: False discovery rate
GO: Gene Ontology
GAPDH: Glyceraldehyde phosphate dehydrogenase
IFN-α: Interferon-α
IFN-γ: Interferon-γ
IL-10: Interleukin-10
KEGG: Kyoto Encyclopedia of Genes and Genomes
KLRG1: Killer cell lectin-like receptor subfamily G member 1
MCAO: Middle cerebral artery occlusion
miRNA: MicroRNA
mRNA: Messenger RNA
mNSS: Modified neurological severity score
MRI: Magnetic resonance imaging
NK cell: Natural killer cell
NKG2A: Natural killer group 2, member A
NKG2D: Natural killer group 2, member D
PBS: Phosphate-buffered saline
STAT1: Signal transducer and activator of transcription 1
Sp1: Specificity protein 1
TGF-β: Transforming growth factor-β
UTR: Untranslated region
