# Supplementary material for: miR-1224 contributes to ischemic stroke-mediated natural killer cell dysfunction by targeting Sp1 signaling
Source: J Neuroinflammation. 2021 Jun 12;18:133. doi: 10.1186/s12974-021-02181-4 (PMC8196447; doi:10.1186/s12974-021-02181-4)
Supplement: Supplementary file 1 — Additional file 1: Supplementary Figure 1. The effect of miR-1224 on the phenotype and function of NK cells in the brain and periphery after ischemia and reperfusion. After treatment with miR-1224 mimics or inhibitor or a negative control, NK cells were transferred intravenously into NPG mice before MCAO. Cells were isolated from the spleen and brain at the indicated time points after surgery. A-C. Flow cytometry plots and summarized results show the influence of miR-1224 on NK cells in the spleen (B) and brain (C) in the acute stage of ischemic stroke, including inhibitory receptors on NK cells (NKG2A and KLRG1), activating receptors on NK cells (NKG2D), and the cytotoxic function of NK cells (perforin). [file 12974_2021_2181_MOESM1_ESM.docx]

**
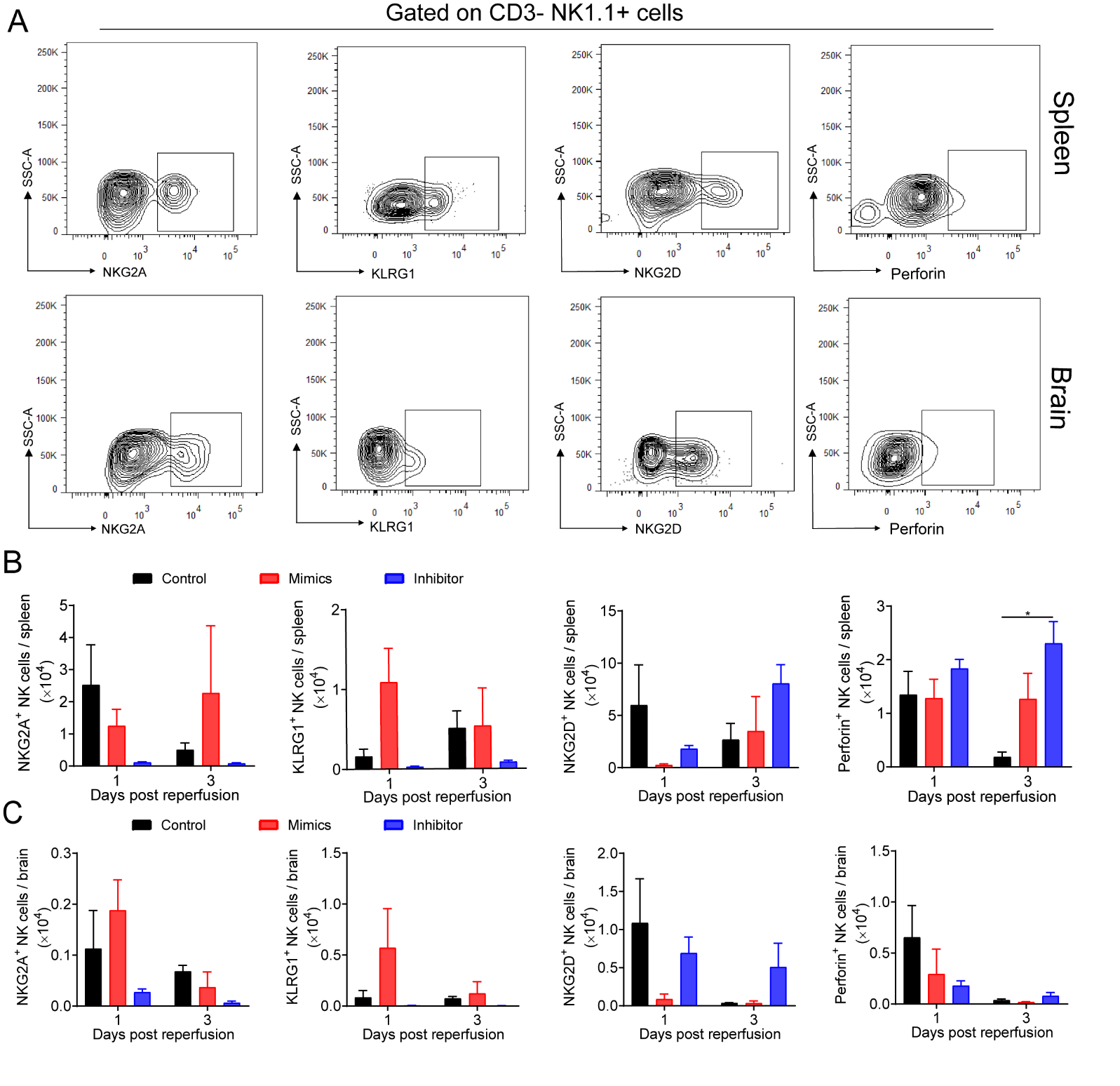
**

**Supplementary Figure 1. The effect of miR-1224 on the phenotype and function of NK cells in the brain and periphery after ischemia and reperfusion.** After treatment with miR-1224 mimics or inhibitor or a negative control, NK cells were transferred intravenously into NPG mice before MCAO. Cells were isolated from the spleen and brain at the indicated time points after surgery. **A-C.** Flow cytometry plots and summarized results show the influence of miR-1224 on NK cells in the spleen **(B)** and brain **(C)** in the acute stage of ischemic stroke, including inhibitory receptors on NK cells (NKG2A and KLRG1), activating receptors on NK cells (NKG2D), and the cytotoxic function of NK cells (perforin)
