# Supplementary material for: miR-1224 contributes to ischemic stroke-mediated natural killer cell dysfunction by targeting Sp1 signaling
Source: J Neuroinflammation. 2021 Jun 12;18:133. doi: 10.1186/s12974-021-02181-4 (PMC8196447; doi:10.1186/s12974-021-02181-4)
Supplement: Supplementary file 2 — Additional file 2: Supplementary Table 1. miR-1224 inhibits the activation and cytotoxicity of splenic NK cells after ischemic stroke. Flow cytometry was used to analyze the number of functional markers in NK cells. Data are presented as the mean ± SEM. *p < 0.05, **p < 0.01 in mimics versus control; #p < 0.05, ##p < 0.01 in inhibitor versus control; &p < 0.05, &&p < 0.01 in inhibitor versus mimics. Supplementary Table 2. miR-1224 is involved in the alteration of brain-infiltrating NK cells after MCAO. Splenic NK cells isolated from wild-type mice were treated with an miR-1224 control, mimic or inhibitor plasmid. After transfection, NK cells were transferred intravenously into NPG mice before MCAO. The table shows the NK cell counts and the expression of functional markers of NK cells in the ischemic brain. Data are expressed as the mean ± SEM. *p < 0.05, **p < 0.01 in mimics versus control. Supplementary Table 3. miR-1224 negatively regulates NK cell function by modulating Sp1. Flow cytometry shows splenic NK cell counts and functional markers (CD69 and IFN-γ) of wild-type and Sp1-/- mice subjected to MCAO. Data were presented as the mean ± SEM. *p < 0.05 in WT/inhibitor versus WT/vehicle; #p < 0.05 in WT/inhibitor versus Sp1-/-/inhibitor. Supplementary Table 4. miR-1224 negatively regulates NK cell function by modulating Sp1. Flow cytometry shows brain-infiltrating NK cell counts and functional markers (CD69 and IFN-γ) in wild-type and Sp1-/- mice subjected to MCAO. Data and presented as the mean ± SEM. [file 12974_2021_2181_MOESM2_ESM.docx]

**
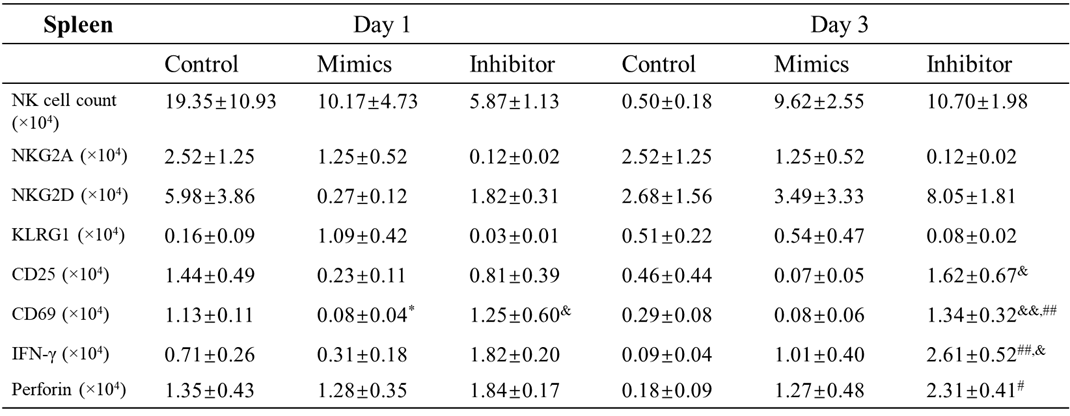
**

**Supplementary Table 1. miR-1224 inhibits the activation and cytotoxicity of splenic NK cells after ischemic stroke.** Flow cytometry was used to analyze the number of functional markers in NK cells. Data are presented as the mean ± SEM. *p<0.05, **p<0.01 in mimics versus control; #p<0.05, ##p<0.01 in inhibitor versus control; &p<0.05, &&p<0.01 in inhibitor versus mimics.


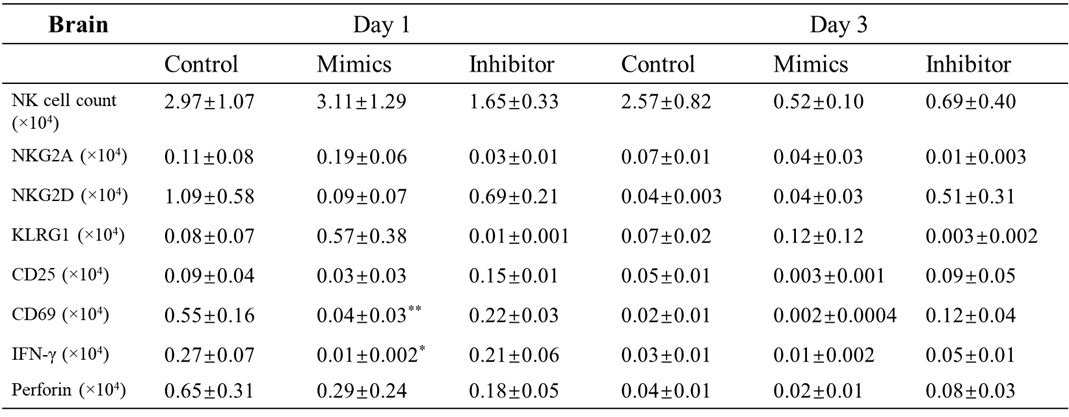


**Supplementary Table 2. miR-1224 is involved in the alteration of brain-infiltrating NK cells after MCAO.** Splenic NK cells isolated from wild-type mice were treated with an miR-1224 control, mimic or inhibitor plasmid. After transfection, NK cells were transferred intravenously into NPG mice before MCAO. The table shows the NK cell counts and the expression of functional markers of NK cells in the ischemic brain. Data are expressed as the mean ± SEM. *p<0.05, **p<0.01 in mimics versus control.

**
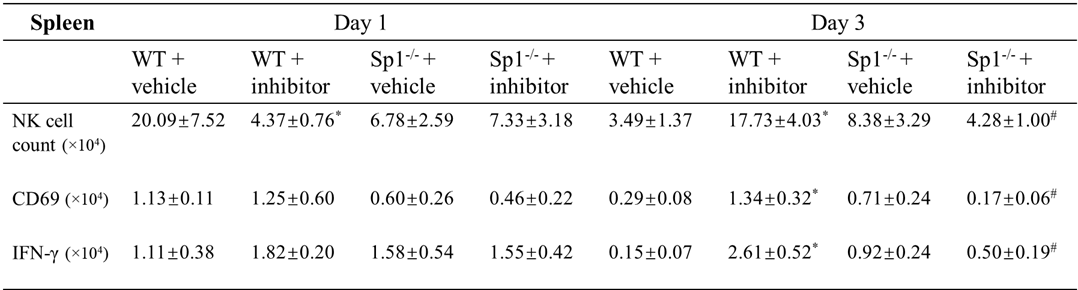
**

**Supplementary Table 3. miR-1224 negatively regulates NK cell function by modulating Sp1.** Flow cytometry shows splenic NK cell counts and functional markers (CD69 and IFN-γ) of wild-type and Sp1^-/-^ mice subjected to MCAO. Data were presented as the mean ± SEM. *p<0.05 in WT/inhibitor versus WT/vehicle; #p<0.05 in WT/inhibitor versus Sp1^-/-^/inhibitor.


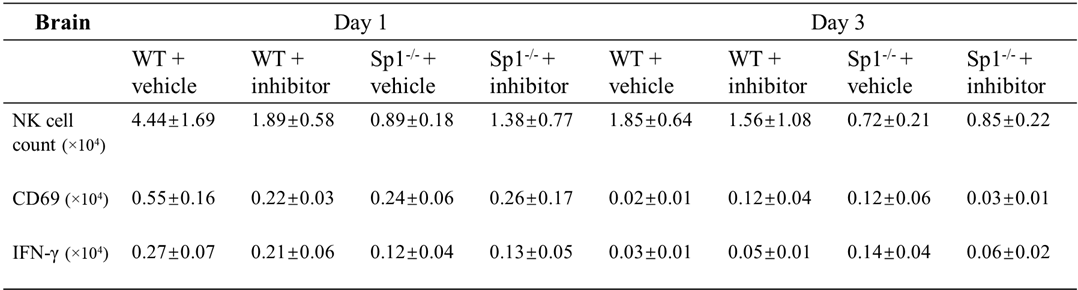


**Supplementary Table 4. miR-1224 negatively regulates NK cell function by modulating Sp1.** Flow cytometry shows brain-infiltrating NK cell counts and functional markers (CD69 and IFN-γ) in wild-type and Sp1^-/-^ mice subjected to MCAO. Data and presented as the mean ± SEM.
